# Supplementary material for: Intriguing role of water in protein-ligand binding studied by neutron crystallography on trypsin complexes
Source: Nat Commun. 2018 Sep 3;9:3559. doi: 10.1038/s41467-018-05769-2 (PMC6120877; doi:10.1038/s41467-018-05769-2)
Supplement: Supplementary file 1 — Supplementary Information [file 41467_2018_5769_MOESM1_ESM.pdf]

Intriguing Role of Water in Protein-Ligand Binding Studied by Neutron Crystallography on Trypsin Complexes

J. Schiebel et al.

Supplementary Figures

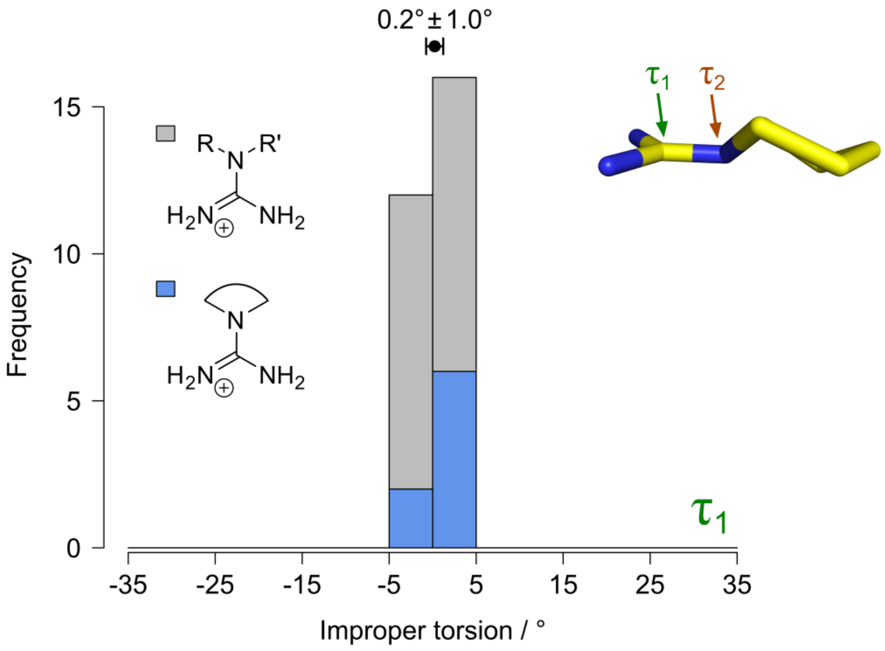

Supplementary Figure 1. CSD search concerning the planarity of guanidino groups. This planarity is quantified by the improper torsion τ_1 (see top right corner and Figure 3). The gray and blue bars represent compounds with two non-H substituents that are separated or linked by a cyclic bridge, respectively. Mean and standard deviation are given above the bars. H-substituted guanidines have been omitted from the CSD search intentionally because X-ray crystallographic coordinates of H-atoms are error-prone. The CSD search, thus, resulted in the limited number of N = 28 hits. The distribution of τ_2 values is shown in Figure 3.

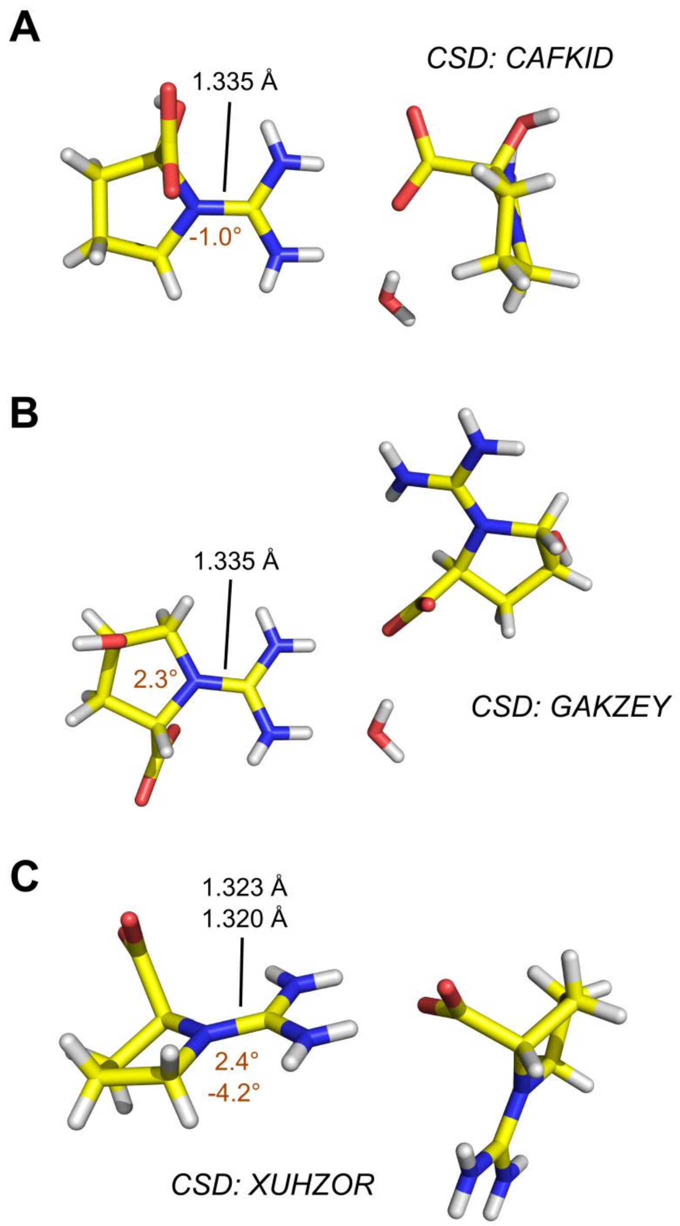

Supplementary Figure 2. Small-molecule X-ray structures of 5-membered guanidino-group containing rings. Together with 6-membered analogs, the structures (Panel A, B, C) represent the subset of the CSD search highlighted in blue in Figure 3F and Supplementary Figure 1. The improper torsion angle characterizing the planarity of the ring nitrogen and the three surrounding carbons as well as the distance between the pyrrolidine nitrogen and the attached guanidino carbon atom are given. For the structure with the CSD-code XUHZOR (Panel C) two values are shown for the improper torsion and bond length since two molecules are present per asymmetric unit of the crystal structure.[1]

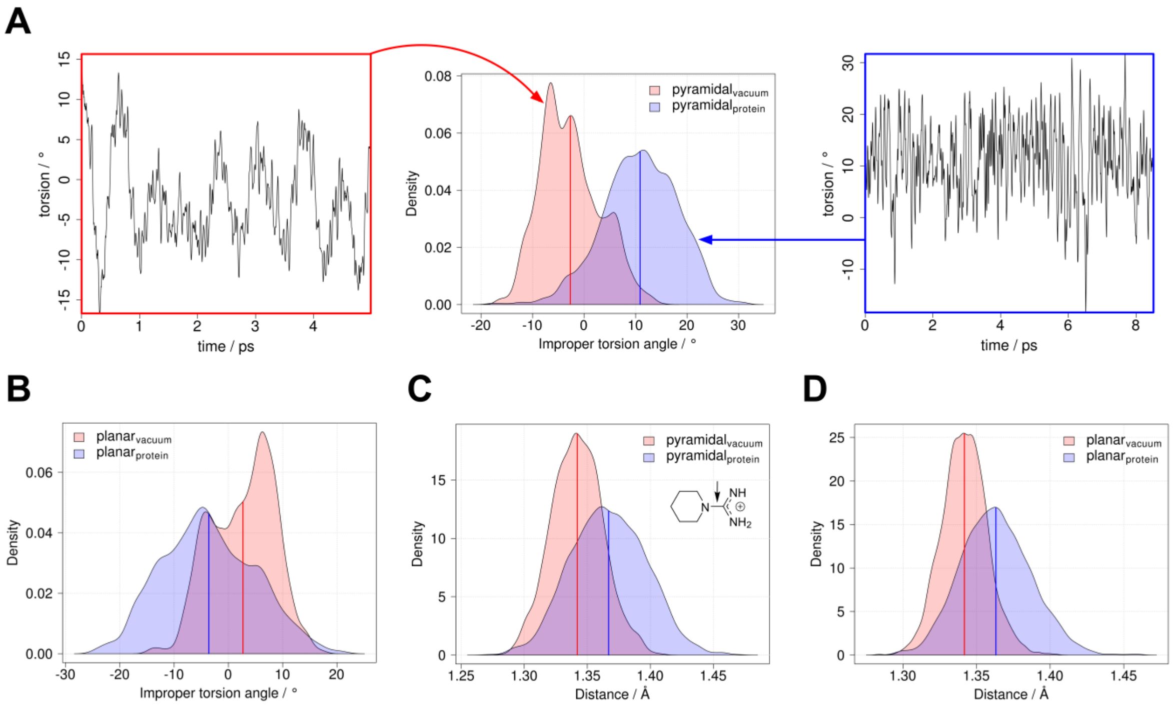

Supplementary Figure 3. Short MD simulations in the absence and presence of trypsin. Protein-free simulations are highlighted in pink while simulations in the presence of trypsin are represented by the blue color. Simulation length was between 5.0 and 8.5 ps. The ligand and selected protein residues have been treated quantum-mechanically. Simulations were started from either the pyramidal (A and C) or planar configuration (B and D) of *N*-amidinopiperidine as present in the 295 K X-ray structure. The plots depict the kernel density estimations of the piperidine N improper torsion angle (A and B) and the distance between the piperidine N and the attached amidino carbon (C and D). Mean values are highlighted by vertical lines and the MD time course is exemplarily shown in panel A.

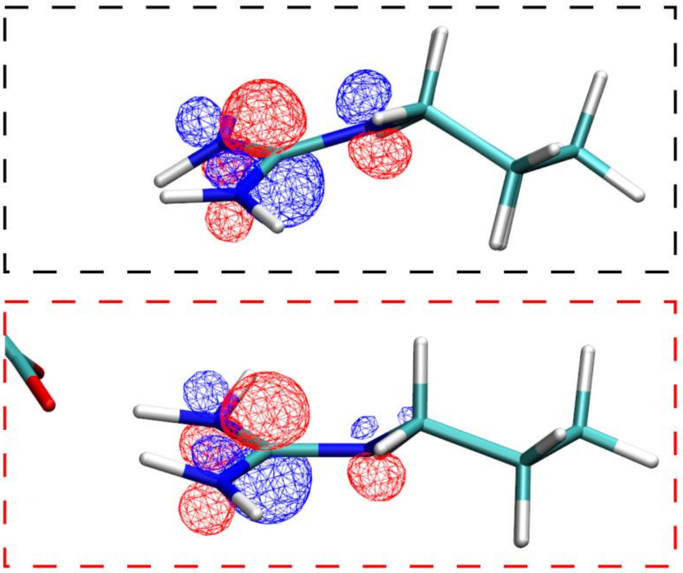

Supplementary Figure 4. Prerequisite for the formation of an *N*-amidinopiperidine conformer pyramidalized at the piperidine nitrogen. Structures resulting from unrestrained geometry optimizations of the free *N*-amidinopiperidine and of an *N*-amidinopiperidine: aspartate complex are shown in the top and bottom boxes, respectively. Both DFT calculations were started from the pyramidal form of *N*-amidinopiperidine on the basis of the 295 K X-ray structure (characterized by an improper torsion angle of 15.7°), which resulted in a planar form for the isolated molecule (improper torsion of 1.2°, top black box) while the pyramidalization was maintained in the presence of an aspartate counter-ion (improper torsion of 17.8°, bottom red box). The LUMO resulting from the calculation with *N*-amidinopiperidine and the corresponding LUMO+1 of the geometry-optimized *N*-amidinopiperidine:aspartate system are shown as meshes.

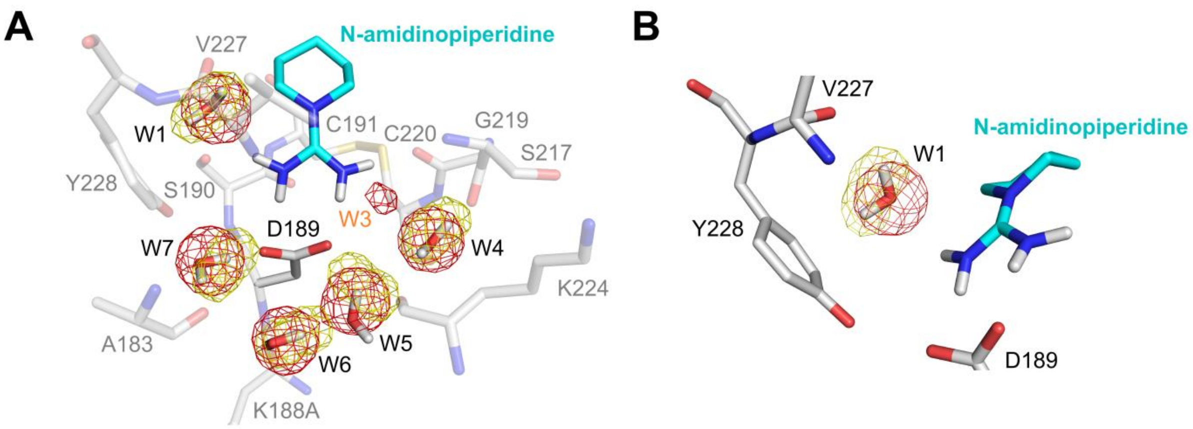

Supplementary Figure 5. Comparison of water orientations close to Asp189 between an MD simulation and the XN structure of the trypsin:*N*-amidinopiperidine complex. Occupancy maps for water oxygen and hydrogen atoms are shown at the 55% level as red and yellow meshes, respectively. While the ligand and selected protein residues are shown (Panel A) in stick representation as present in the first frame of the MD trajectory following the equilibration phase, water molecule stick models were derived from a superimposition of the trypsin:*N*-amidinopiperidine XN structure onto this frame. Panel B provides a more detailed view of water molecule W1 located on top of Tyr228.

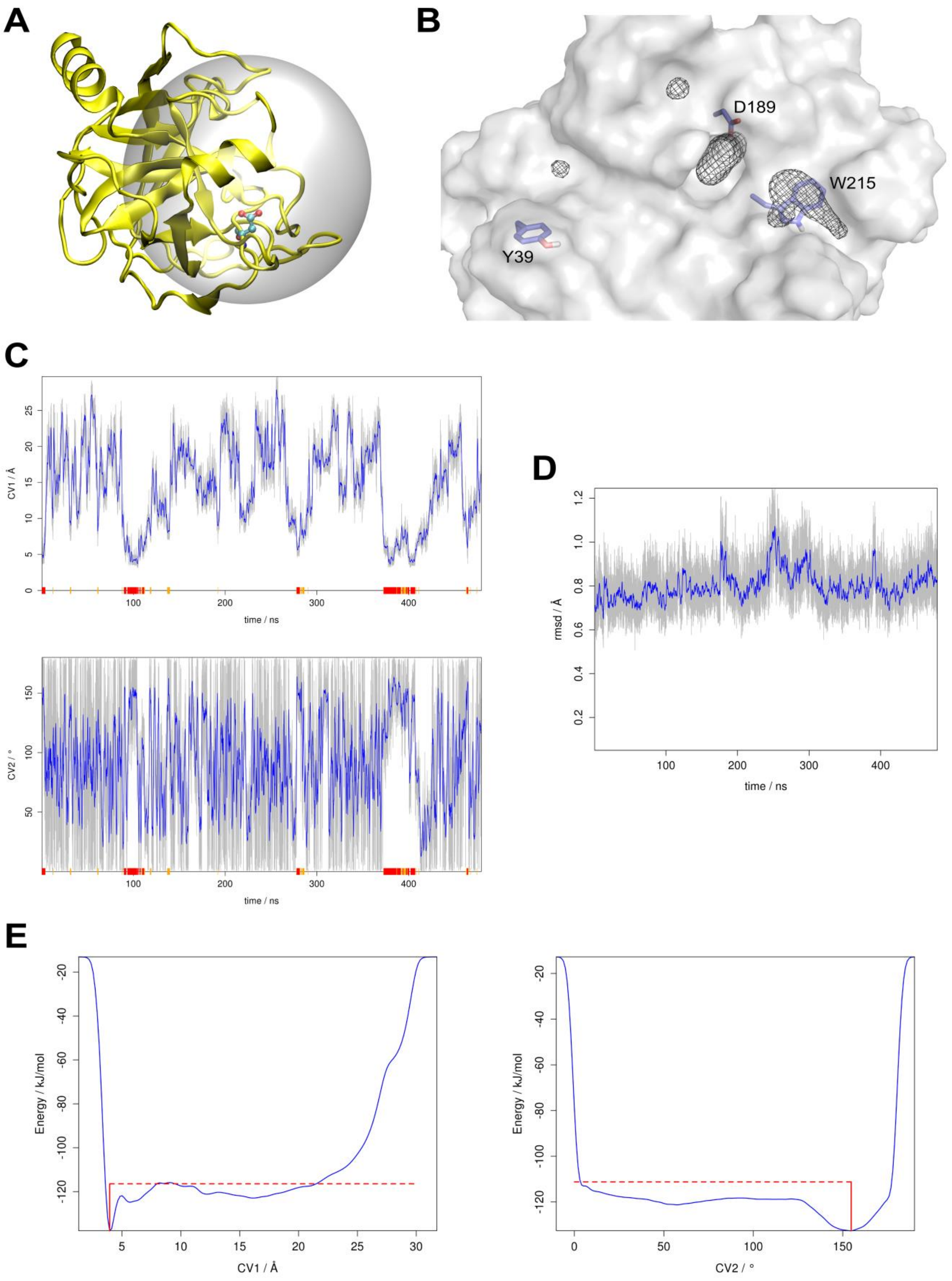

Supplementary Figure 6. Characteristics of the metadynamics run starting from the trypsin:*N*-amidinopiperidine complex. (A) Simulation confinement region surrounding the S_1 pocket of trypsin (for details, please refer to the Experimental Section). In case the ligand's center-of-mass left the region represented by the silver sphere during the simulation, a

restraining potential was switched on to keep the ligand within this volume. Asp189 is shown as cyan ball-and-stick model. (B) Description of those regions *N*-amidinopiperidine visits most frequently. Trypsin is represented by the transparent gray surface model with the view onto the horizontally-oriented binding cleft of this protease. An occupancy map for the guanidino carbon of *N*-amidinopiperidine is depicted at the 1.8% level in gray. While Asp189 defines the location of the S_1 pocket, Tyr39 and Trp215 are located close to the S_2' and $S_{3/4}$ pockets, respectively. (C) Development of the CV1 and CV2 parameters along the simulation. While values every 10 ps are plotted in gray, the blue line represents the sliding average over 1 ns intervals. States in which the ligand can be considered to be bound as evaluated by CV1 ≤ 5 Å and 120° < CV2 < 180° and corresponding to minimum a in Figure 5A, are marked in red on the x-axis. Similarly, states in which the ligand is almost bound (5 Å < CV1 ≤ 7 Å and 120° < CV2 < 180°, minimum b in Figure 5A) are highlighted in orange. (D) Stability of rmsd values during the simulation. The backbone rmsd extracted every 10 ps and calculated following a least-squares fit of all backbone atoms onto the first frame are plotted in gray, while the sliding average over 1 ns intervals is overlaid in blue. (E) Projection of the free energy surface onto CV1 and CV2, respectively. The Gibbs free energy of binding as derived from our direct ITC titration experiments performed in three different buffers is shown in red as an experimental reference (buffer-corrected value from Supplementary Table 2). Please note that the steep increase in energy at large CV1 values originates from the applied restraining potential (see panel A).

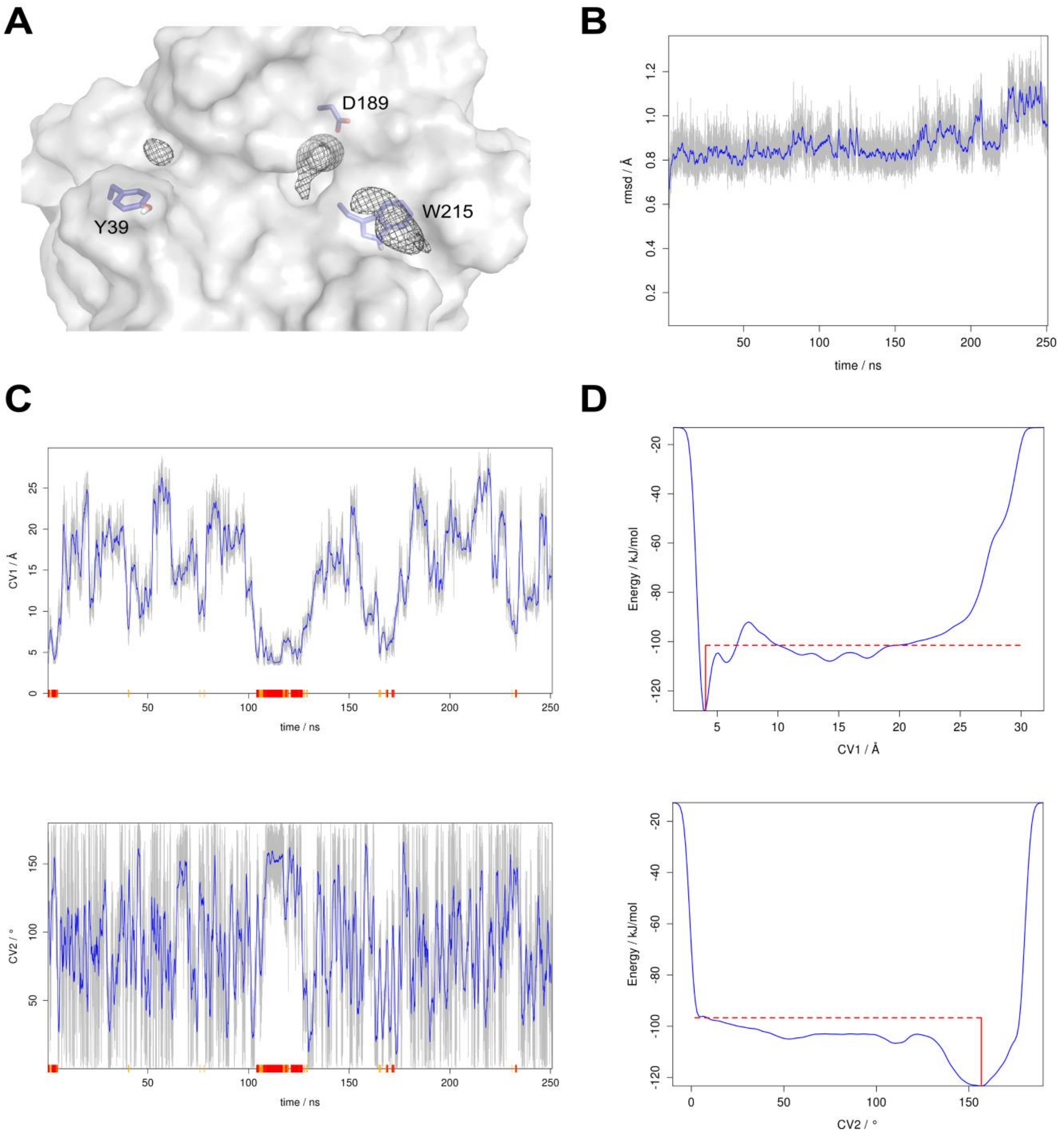

Supplementary Figure 7. Characteristics of the metadynamics run starting from the trypsin:benzamidine complex. (A) Description of those regions benzamidine visits most frequently. Trypsin is represented by the transparent gray surface model with the view onto the horizontally-oriented binding cleft of this protease. An occupancy map for the amidino-group carbon of benzamidine is depicted at the 1.8% level in gray. While Asp189 defines the location of the S_1 pocket, Tyr39 and Trp215 are located close to the S_2' and $S_{3/4}$ pockets, respectively. (B) Stability of rmsd values during the simulation. The backbone rmsd extracted every 10 ps and calculated following a least-squares fit of all backbone atoms onto the first frame are plotted

in gray, while the sliding average over 1 ns intervals is overlaid in blue. (C) Development of the CV1 and CV2 parameters along the simulation. While values every 10 ps are plotted in gray, the blue line represents the sliding average over 1 ns intervals. States in which the ligand can be considered to be bound as evaluated by CV1 ≤ 5 Å and 125° < CV2 < 180° and corresponding to minimum a in Figure 5B, are marked in red on the x-axis. Similarly, states in which the ligand is almost bound (5 Å < CV1 ≤ 7 Å and 100° < CV2 < 125°, minimum b in Figure 5B) are highlighted in orange. (D) Projection of the free energy surface onto CV1 and CV2, respectively. The Gibbs free energy of binding as derived from our direct ITC titration experiments performed in three different buffers is shown in red as an experimental reference (buffer-corrected value from Supplementary Table 2). Please note that the steep increase in energy at large CV1 values originates from the applied restraining potential (see Supplementary Figure 6A).

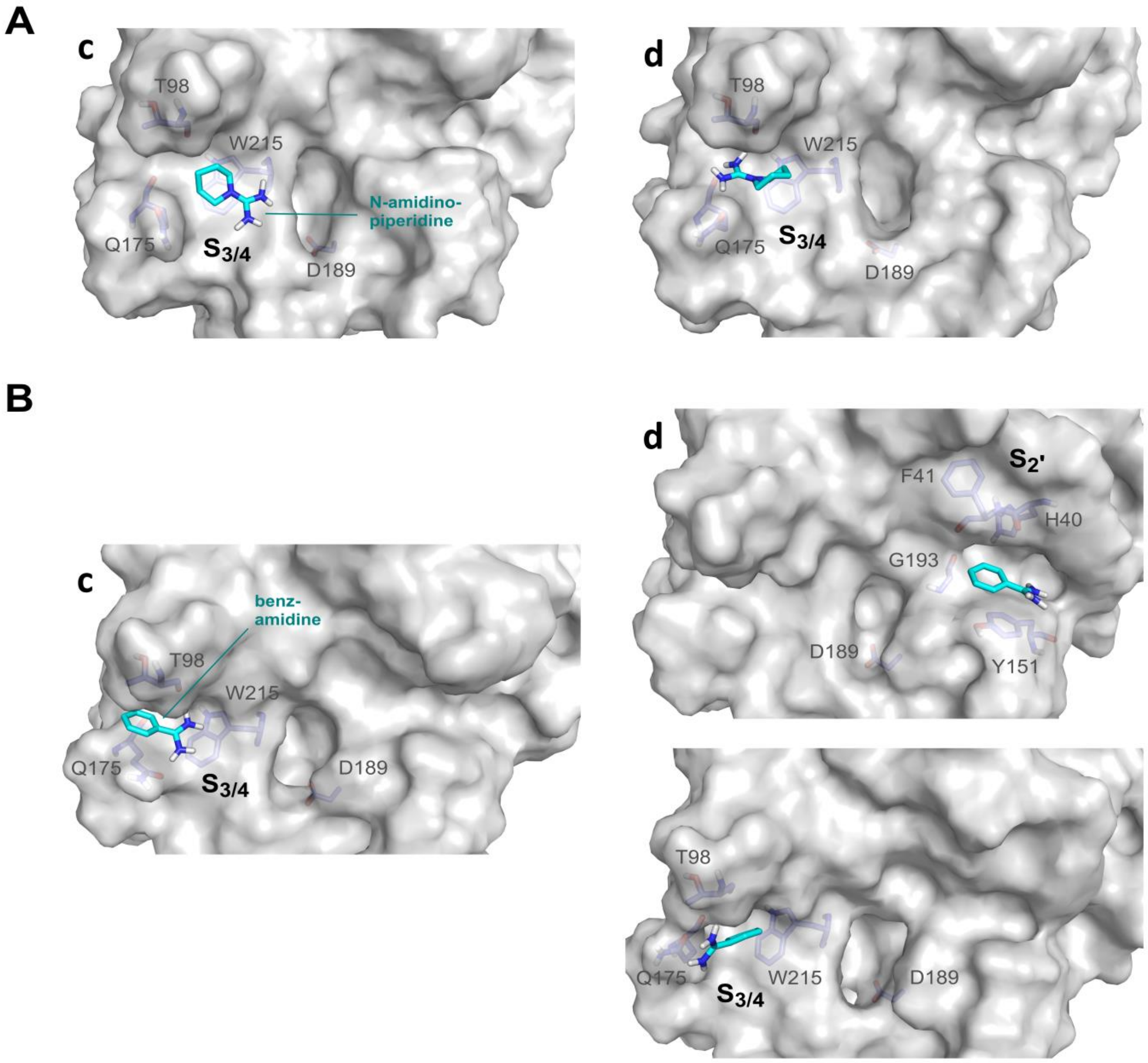

Supplementary Figure 8. Representative structural snapshots corresponding to minor energy minima on the free energy surface of trypsin:inhibitor complexes. These minima are shown for the inhibitors *N*-amidinopiperidine (A) and benzamidine (B) and labeled with the letters c and d in the above figure as well as in the corresponding FES presented in Figure 5. Ligands are shown in cyan. For benzamidine, minimum d does not correspond to an individual state but rather can be linked to a situation where benzamidine is either bound in the S_2' (top) or $S_{3/4}$-pocket (bottom).

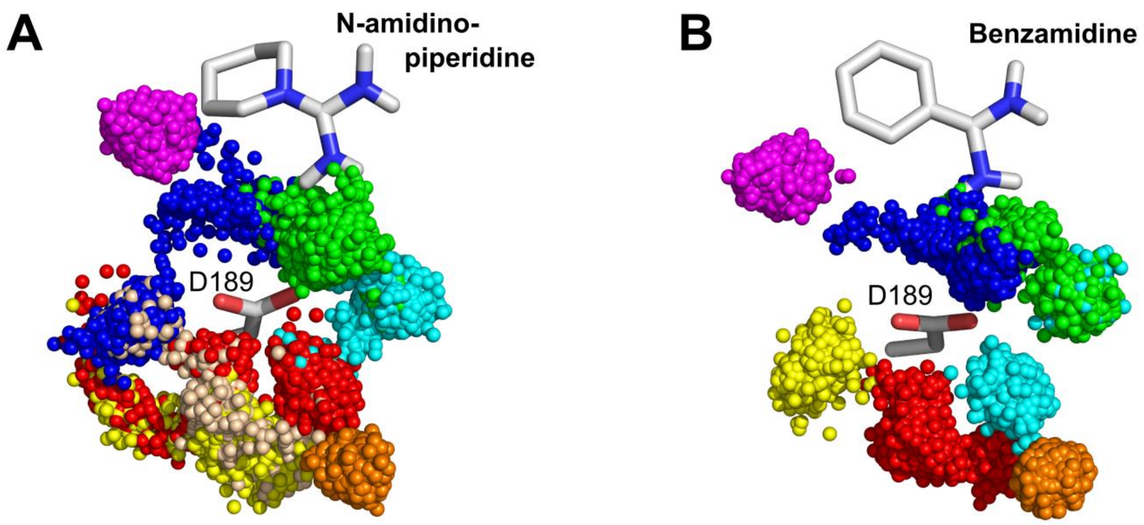

Supplementary Figure 9. Mobility of waters during the dissociation of ligands from trypsin. For the trypsin:*N*-amidinopiperidine (A) and trypsin:benzamidine complexes (B), individual water molecules surrounding Asp189 during a selected unbinding event along the metadynamics trajectory are represented by spheres in discrete colors at 10 ps intervals between the bound and a situation where the ligand is separated from Asp189 by one solvation layer. The latter state is represented by the stick model of Asp189 and *N*-amidinopiperidine. Supplementary Movies 1 and 3 describe the same unbinding events.

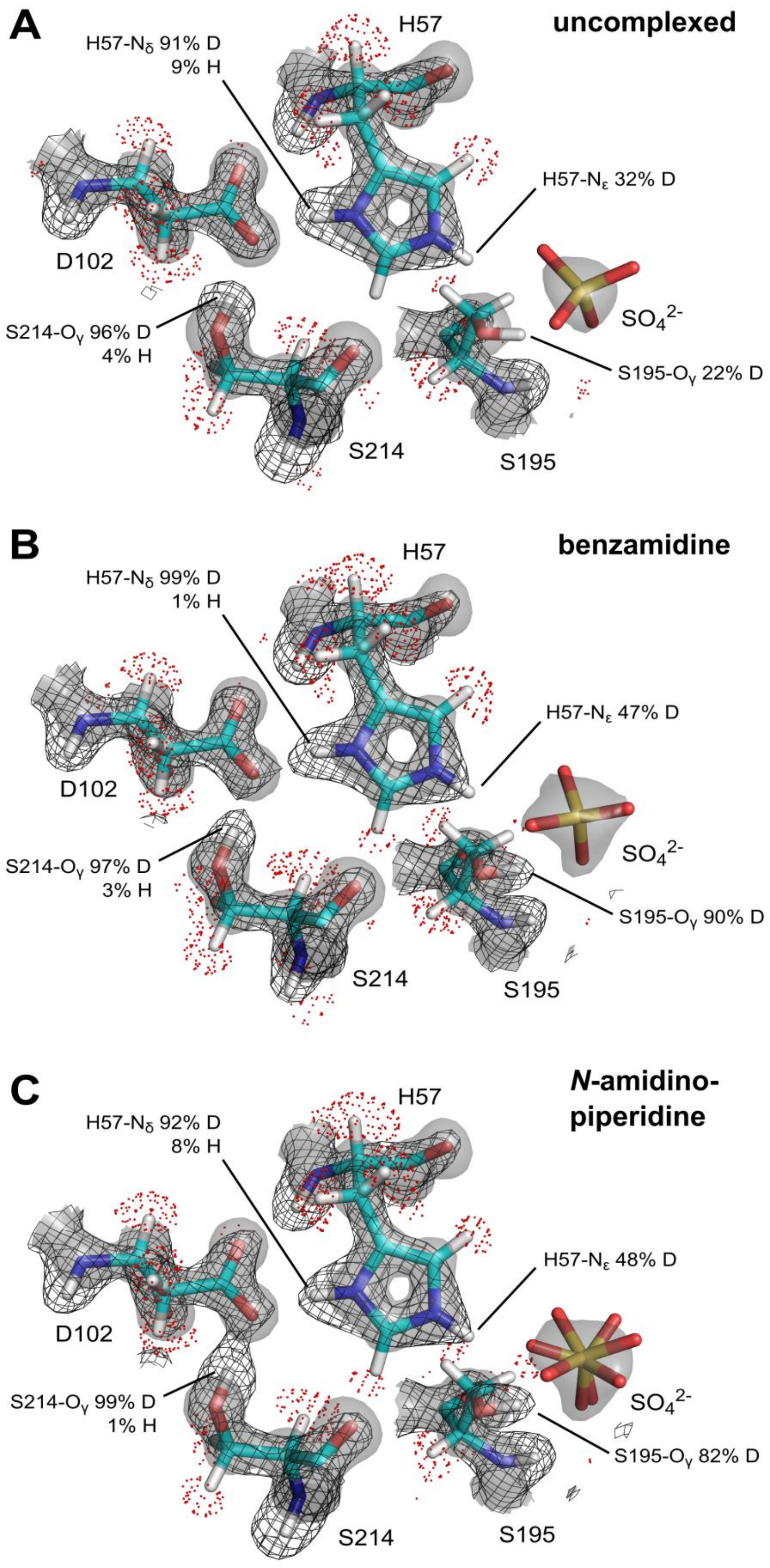

Supplementary Figure 10. Modeling of the catalytic triad and Ser214 in the trypsin XN structures. During refinement of the XN structures of uncomplexed trypsin (A), trypsin:benzamidine (B) and trypsin:*N*-amidinopiperidine (C), nuclear density maps indicated

that the His57-N$_\delta$ and Ser214-O$_\gamma$ atoms are deuterated to a large extent. An H/D occupancy refinement was performed at these sites because they are not fully solvent-exposed and involved in H-bonding. The resulting occupancies of H and D-atoms are indicated in the figure. In contrast, much lower nuclear density peaks were observed for the His57-N$_\varepsilon$ and Ser195-O$_\gamma$ atoms indicating either hindered H/D exchange or incomplete protonation. Since these atoms are directly solvent-exposed and not involved in a highly stable H-bond that might explain a slow H/D exchange (all analyzed crystals were soaked in deuterated mother liquor for at least two weeks), we expected that both atoms are fully H/D exchanged. Accordingly, we modeled only a deuteron at these sites and refined its occupancy. The results, which are highlighted in the figure, indicate that His57-N$_\varepsilon$ is partially protonated leading to a positive charge on this amino acid while Ser195 might partially exist in a deprotonated and thus nucleophilic, substrate-reactive state. A sulfate ion, which originates from the crystallization solution, was found to be present close to Ser195 and His57 albeit with high mobility and/or partial occupancy. The sulfate might mimic the negative charge of the oxyanion generated during substrate turnover. The above-described findings are supported by the 2mF$_o$-DF$_c$ electron and nuclear density maps shown at the 2σ level as transparent gray surface and gray mesh, respectively. While deuterons appear as positive peaks in the 2mF$_o$-DF$_c$ nuclear density map, hydrogens result in negative peaks due to the negative neutron scattering length of the H atom. In the figure, the 2mF$_o$-DF$_c$ nuclear density map is therefore additionally shown at the -2σ level in the form of red dots.

Supplementary Tables

Supplementary Table 1. Inhibition of trypsin and thrombin by derivatives of *N*-amidinopiperidine and benzamidine (arithmetic mean ± standard deviation from measurements performed at least in triplicate).

	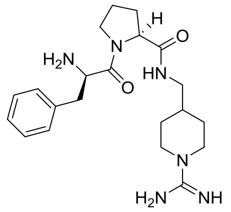 D-Phe-Pro-NHCH$_2$-N-amidinopiperidine (**1**)		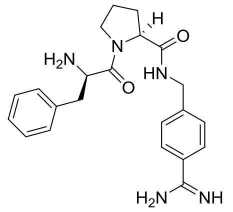 D-Phe-Pro-NHCH$_2$-benzamidine (**2**)	
	trypsin	*thrombin*	*trypsin*	*thrombin*
K_i [nM] [a]	1959 ± 264	4.51 ± 0.25	15.2 ± 0.2	0.36 ± 0.03

[a] determined using a fluorescence assay

Supplementary Table 2. Thermodynamic parameters for *N*-amidinopiperidine and benzamidine binding to trypsin (arithmetic mean ± standard deviation from measurements performed at least in triplicate).

**Method**	**Buffer**	**Ligand**	**K_d [µM]**	**$\Delta G°$ [kJ mol^{-1}]**	**$\Delta H°$ [kJ mol^{-1}]**	**$-T\Delta S°$ [kJ mol^{-1}]**
Direct titration	HEPES	*N*-amidino-piperidine	143 ± 11	-22.0 ± 0.2	-3.8 ± 0.2	-18.2 ± 0.3
		Benzamidine	20.7 ± 2.8	-26.7 ± 0.3	-11.7 ± 0.1	-15.0 ± 0.3
	Tricine	*N*-amidino-piperidine	184 ± 9	-21.3 ± 0.1	-6.9 ± 0.2	-14.4 ± 0.2
		Benzamidine	22.6 ± 0.4	-26.5 ± 0.04	-15.9 ± 0.6	-10.6 ± 0.6
	Tris	*N*-amidino-piperidine	228 ± 3	-20.8 ± 0.04	-12.9 ± 0.2	-7.9 ± 0.2
		Benzamidine	23.1 ± 0.3	-26.5 ± 0.03	-19.9 ± 0.4	-6.6 ± 0.4
	Buffer-corrected	*N*-amidino-piperidine	185 [a]	-21.4 [a]	3.8 [b]	-25.1 [c]
		Benzamidine	22.2 [a]	-26.6 [a]	-5.6 [b]	-21.0 [c]
Displace-ment titration	HEPES	*N*-amidino-piperidine	366 ± 105	-19.6 ± 0.7	-10.9 ± 1.0	-8.7 ± 1.2
		Benzamidine	23.8 ± 5.3	-26.4 ± 0.6	-16.1 ± 0.7	-10.2 ± 0.9

[a] $\Delta G°$ and K_d are given as the average over the results determined in the three different buffers

[b] $\Delta H°$ values determined in the three chosen buffer systems have been plotted vs. the ionization enthalpy of each buffer (HEPES[2]: 21.01 kJ mol^{-1}, Tricine[2]: 31.97 kJ mol^{-1}, Tris[3]: 47.53 kJ mol^{-1}); the intercept of the associated linear interpolation function corresponding to the enthalpy of the binding reaction in a hypothetical buffer with an ionization enthalpy of 0 kJ mol^{-1} has been reported as buffer-corrected value in the table; the slope of the interpolation function that reflects the number of protons transferred from the buffer onto trypsin upon complex formation accounted for -0.35 and -0.31 in the case of *N*-amidinopiperidine and benzamidine, respectively

[c] $-T\Delta S°$ has been calculated as the difference between $\Delta G°$ and $\Delta H°$

Supplementary Table 3. Thermodynamic parameters for *N*-amidinopiperidine and benzamidine derivatives binding to trypsin (see also Supplementary Table 1, arithmetic mean ± standard deviation from measurements performed at least in triplicate).

**Buffer**	**Ligand**	**K_d [μM]**	**$\Delta G°$ [kJ mol^{-1}]**	**$\Delta H°$ [kJ mol^{-1}]**	**$-T\Delta S°$ [kJ mol^{-1}]**
HEPES	Compound **1**	3.7 ± 0.2	-31.0 ± 0.2	-19.4 ± 0.3	-11.6 ± 0.3
	Compound **2**	0.038 ± 0.007	-42.4 ± 0.4	-35.5 ± 0.7	-6.9 ± 0.8
Tricine	Compound **1**	3.3 ± 0.3	-31.3 ± 0.2	-18.1 ± 0.5	-13.2 ± 0.5
	Compound **2**	0.034 ± 0.003	-42.6 ± 0.2	-31.6 ± 0.4	-11.0 ± 0.4
Tris	Compound **1**	3.7 ± 0.6	-31.0 ± 0.4	-16.3 ± 0.6	-14.7 ± 0.7
	Compound **2**	0.031 ± 0.005	-42.8 ± 0.4	-30.9 ± 0.4	-12.0 ± 0.6
Buffer-corrected	Compound **1**	3.6 [a]	-31.1 [a]	-21.8 [b]	-9.3 [c]
	Compound **2**	0.034 [a]	-42.6 [a]	-38.2 [b]	-4.4 [c]

[a] $\Delta G°$ and K_d are given as the average over the results determined in the three different buffers

[b] $\Delta H°$ values determined in the three chosen buffer systems have been plotted vs. the ionization enthalpy of each buffer (HEPES[2]: 21.01 kJ mol^{-1}, Tricine[2]: 31.97 kJ mol^{-1}, Tris[3]: 47.53 kJ mol^{-1}); the intercept of the associated linear interpolation function corresponding to the enthalpy of the binding reaction in a hypothetical buffer with an ionization enthalpy of 0 kJ mol^{-1} has been reported as buffer-corrected value in the table; the slope of the interpolation function that reflects the number of protons transferred from the buffer onto trypsin upon complex formation accounted for 0.12 and 0.17 in the case of compound **1** and **2**, respectively

[c] $-T\Delta S°$ has been calculated as the difference between $\Delta G°$ and $\Delta H°$

Supplementary Table 4. Neutron diffraction data collection and refinement statistics.[a]

**Trypsin ligand**	Apo	*N*-amidinopiperidine	Benzamidine
**Data-collection temperature (K)**	295	295	295
**PDB code**	5MNZ	5MO2	5MO0
**Data collection and processing** [b]			
Wavelength (Å)	2.672	2.676	2.673
Beamline	FRM II, BIODIFF	FRM II, BIODIFF	FRM II, BIODIFF
Detector	Maatel neutron image plate	Maatel neutron image plate	Maatel neutron image plate
Space group	$P2_12_12_1$	$P2_12_12_1$	$P2_12_12_1$
Cell dimensions			
a, b, c (Å)	55.0, 58.5, 67.5	54.9, 58.6, 67.5	54.9, 58.7, 67.5
α, β, γ (°)	90.0, 90.0, 90.0	90.0, 90.0, 90.0	90.0, 90.0, 90.0
Resolution range (Å)	50.0-1.45 (1.48-1.45)	50.0-1.50 (1.53-1.50)	50.0-1.50 (1.53-1.50)
No. of unique reflections	35542 (1415)	31665 (1869)	31713 (1610)
Average redundancy	3.0 (2.7)	3.1 (2.6)	2.4 (1.7)
R_{merge} (%)	12.6 (47.4)	11.0 (45.4)	7.1 (26.7)
Completeness (%)	90.2 (72.8)	88.7 (79.9)	89.4 (69.6)
$\langle I/\sigma(I)\rangle$	6.9 (2.1)	7.5 (2.2)	10.2 (3.2)
CC1/2	(0.58)	(0.68)	(0.79)
**Refinement**			
Resolution range (Å)	21.3 - 1.45	22.1 - 1.50	25.4 - 1.50
No. of reflections (total / free)	35510 / 1784	31623 / 1587	31693 / 1573
R_{cryst} (%)	16.9	16.1	14.7
R_{free} (%)	20.1	20.0	18.5
No. of refined protein residues	223	223	223
Protein hydrogen atoms visible (%)	87	88	89
No. of ligand non-hydrogen atoms	-	9	9
No. of ligand hydrogen atoms	-	14	9
No. of other ligand atoms [c]	1	2	1
No. of water molecules	136	137	144
No. of visible water hydrogens	202	206	225
RMSD, bond lengths (Å)	0.004	0.004	0.004
RMSD, bond angles (°)	0.9	0.9	0.9
Ramachandran plot (%) [d]			
Most favored / favored	87.8 / 98.2	87.8 / 98.6	87.8 / 98.6
Additionally allowed / allowed	12.2 / 1.8	12.2 / 1.4	12.2 / 1.4
Generously allowed / -	0.0 / -	0.0 / -	0.0 / -
Disallowed / outliers	0.0 / 0.0	0.0 / 0.0	0.0 / 0.0
Average *B* factors ($Å^2$) [e]			
All protein atoms	13.4	12.1	11.7
Main chain	11.9	10.4	9.7
Side chain	14.0	12.7	12.5
Ligand atoms	-	14.0	12.2
Other ligand atoms [c]	8.9	11.5	7.6
Waters	21.4	19.7	21.4

[a] All crystals used for diffraction experiments have been deuterated before data collection (for details see Experimental Section). Although H and D atoms are distinguished by neutron crystallography, all H and D atoms are referred to as hydrogens in the table.
[b] Values in parenthesis refer to the highest resolution shell
[c] Other ligands are Ca^{2+}
[d] Calculated using PROCHECK[4] and MOLPROBITY[5], respectively; first value indicates PROCHECK results, second MOLPROBITY values as calculated in the PDB validation report
[e] Calculated using MOLEMAN including hydrogen atoms[6]

Supplementary Table 5. X-ray diffraction data collection and refinement statistics (100 K).[a]

**Trypsin ligand**	Apo	N-amidinopiperidine	Benzamidine	D-Phe-Pro-N-amidinopiperidine
**Data-collection temperature (K)**	100	100	100	100
**PDB code**	5MNE	5MNN	5MNG	5MNQ
**Data collection and processing** [b]				
Wavelength (Å)	0.7069	0.7069	0.7069	0.9184
Beamline	DESY, EMBL P14	DESY, EMBL P14	DESY, EMBL P14	BESSY, BL 14.1
Detector	PILATUS 6M	PILATUS 6M	PILATUS 6M	PILATUS 6M
Space group	$P2_12_12_1$	$P2_12_12_1$	$P2_12_12_1$	$P2_12_12_1$
Cell dimensions				
a, b, c (Å)	54.7, 58.3, 66.9	54.7, 58.2, 66.8	54.5, 58.2, 66.8	59.9, 64.1, 69.4
α, β, γ (°)	90.0, 90.0, 90.0	90.0, 90.0, 90.0	90.0, 90.0, 90.0	90.0, 90.0, 90.0
Resolution range (Å)	42.3-1.01 (1.07-1.01)	42.3-0.86 (0.91-0.86)	43.9-0.86 (0.91-0.86)	47.1-1.34 (1.42-1.34)
Wilson B factor ($Å^2$)	9.6	7.5	7.7	12.6
No. of unique reflections	110736 (17211)	179705 (28611)	179771 (28706)	60257 (9453)
Average redundancy	6.6 (6.6)	8.5 (8.4)	6.4 (6.3)	4.2 (4.1)
R_{merge} (%)	3.3 (56.5)	3.5 (57.3)	3.1 (57.2)	5.4 (49.4)
Completeness (%)	97.8 (94.9)	99.1 (98.5)	99.8 (99.4)	98.7 (96.9)
$\langle I/\sigma(I) \rangle$	26.3 (3.3)	27.4 (3.6)	26.6 (3.2)	14.2 (2.3)
CC1/2	1.00 (0.88)	1.00 (0.91)	1.00 (0.88)	1.00 (0.86)
**Refinement**				
Resolution range (Å)	24.7 - 1.01	39.9 - 0.86	24.7 - 0.86	45.4 - 1.34
No. of reflections (total / free)	110662 / 5532	179602 / 8983	179654 / 8986	60252 / 3013
R_{cryst} (%)	10.6	10.0	9.9	13.7
R_{free} (%)	12.5	11.0	10.9	17.1
No. of refined protein residues	223	223	223	223
Protein hydrogen atoms visible (%)	43	55	55	-
No. of ligand atoms	-	9	9 + 5 hydrogens	29
No. of other ligand atoms [c]	21	21	21	32
No. of water molecules	331	318	320	235
No. of visible water hydrogens	14	27	27	-
RMSD, bond lengths (Å)	0.009	0.009	0.009	0.008
RMSD, bond angles (˚)	1.2	1.2	1.2	1.2
Ramachandran plot (%) [d]				
Most favored / favored	86.7 / 98.5	87.2 / 98.6	86.7 / 97.9	88.3 / 97.8
Additionally allowed / allowed	13.3 / 1.5	12.8 / 1.4	12.8 / 2.1	11.2 / 2.2
Generously allowed / -	0.0 / -	0.0 / -	0.0 / -	0.5 / -
Disallowed / outliers	0.0 / 0.0	0.0 / 0.0	0.5 / 0.0	0.0 / 0.0
Average *B* factors ($Å^2$) [e]				
All protein atoms	11.3	8.9	9.1	16.3
Main chain	10.2	8.0	8.3	15.1
Side chain	12.4	9.8	10.0	17.4
Ligand atoms	-	9.3	8.3	18.9
Other ligand atoms [c]	20.2	15.8	20.1	32.8
Waters	26.1	23.0	23.9	30.9

[a] All crystals used for diffraction experiments have been deuterated before data collection (for details see Experimental Section). Since H and D atoms cannot be distinguished by X-ray crystallography, all H and D atoms have been modeled as hydrogens and appear as such in the statistics given in the table.
[b] Values in parenthesis refer to the highest resolution shell
[c] Other ligands are sulfate and Ca^{2+}
[d] Calculated using PROCHECK[4] and MOLPROBITY[5], respectively; first value indicates PROCHECK results, second MOLPROBITY values as calculated in the PDB validation report
[e] Calculated using MOLEMAN excluding hydrogen atoms[6]

Supplementary Table 6. X-ray diffraction data collection and refinement statistics (295 K).[a]

**Trypsin ligand**	Apo	N-amidinopiperidine	Benzamidine
**Data-collection temperature (K)**	295	295	295
**PDB code**	5MNF	5MNO	5MNH
**Data collection and processing** [b]			
Wavelength (Å)	0.7749	0.7749	0.7749
Beamline	DESY, EMBL P14	DESY, EMBL P14	DESY, EMBL P14
Detector	PILATUS 6M	PILATUS 6M	PILATUS 6M
Space group	P212121	P212121	P212121
Cell dimensions			
a, b, c (Å)	55.0, 58.6, 67.6	54.9, 58.6, 67.6	54.9, 58.6, 67.6
α, β, γ (°)	90.0, 90.0, 90.0	90.0, 90.0, 90.0	90.0, 90.0, 90.0
Resolution range (Å)	44.3-0.99 (1.05-0.99)	42.6-0.96 (1.02-0.96)	42.6-0.93 (0.99-0.93)
Wilson B factor (Å^2)	10.4	9.7	9.6
No. of unique reflections	121717 (19424)	132628 (21147)	145727 (23129)
Average redundancy	8.7 (8.3)	8.7 (8.4)	8.6 (8.4)
R_{merge} (%)	3.9 (57.4)	3.8 (56.6)	3.9 (56.5)
Completeness (%)	99.8 (99.5)	99.4 (98.9)	99.5 (98.8)
$\langle I/\sigma(I) \rangle$	27.0 (4.3)	28.4 (4.3)	28.7 (4.5)
CC1/2	1.00 (0.88)	1.00 (0.89)	1.00 (0.88)
**Refinement**			
Resolution range (Å)	19.2 - 0.99	27.5 - 0.96	27.5 - 0.93
No. of reflections (total / free)	121609 / 6080	132539 / 6624	145638 / 7284
R_{cryst} (%)	9.6	9.4	9.4
R_{free} (%)	10.7	10.6	10.1
No. of refined protein residues	223	223	223
Protein hydrogen atoms visible (%)	38	37	37
No. of ligand atoms	-	9	9 + 2 hydrogens
No. of other ligand atoms [c]	6	11	11
No. of water molecules	184	169	181
No. of visible water hydrogens	12	12	13
RMSD, bond lengths (Å)	0.008	0.008	0.008
RMSD, bond angles (˚)	1.1	1.1	1.1
Ramachandran plot (%) [d]			
Most favored / favored	87.8 / 99.2	88.3 / 99.2	88.8 / 98.9
Additionally allowed / allowed	12.2 / 0.8	11.7 / 0.8	11.2 / 1.1
Generously allowed / -	0.0 / -	0.0 / -	0.0 / -
Disallowed / outliers	0.0 / 0.0	0.0 / 0.0	0.0 / 0.0
Average *B* factors (Å^2) [e]			
All protein atoms	13.1	12.5	12.3
Main chain	11.7	11.0	10.9
Side chain	14.8	14.2	14.0
Ligand atoms	-	12.6	12.0
Other ligand atoms [c]	50.5	30.3	28.2
Waters	31.5	30.0	30.5

[a] All crystals used for diffraction experiments have been deuterated before data collection (for details see Experimental Section). Since H and D atoms cannot be distinguished by X-ray crystallography, all H and D atoms have been modeled as hydrogens and appear as such in the statistics given in the table.
[b] Values in parenthesis refer to the highest resolution shell
[c] Other ligands are sulfate and Ca^{2+}
[d] Calculated using PROCHECK[4] and MOLPROBITY[5], respectively; first value indicates PROCHECK results, second MOLPROBITY values as calculated in the PDB validation report
[e] Calculated using MOLEMAN excluding hydrogen atoms[6]

Supplementary Table 7. Joint X-ray/neutron refinement statistics.[a]

**Trypsin ligand**	Apo	N-amidinopiperidine	Benzamidine
**Data-collection temperature (K)**	295	295	295
**PDB code**	5MOP	5MOS	5MOQ
**Data collection and processing**			
Neutron part	see PDB code 5MNZ in Supplementary Table 4	see PDB code 5MO2 in Supplementary Table 4	see PDB code 5MO0 in Supplementary Table 4
X-ray part	see PDB code 5MNF in Supplementary Table 6	see PDB code 5MNO in Supplementary Table 6	see PDB code 5MNH in Supplementary Table 6
**Refinement (neutron part)**			
Resolution range (Å)	21.3 - 1.45	22.2 - 1.50	25.4 - 1.50
No. of reflections (total / free)	35510 / 1784	31623 / 1587	31693 / 1573
$R_{cryst,neutron}$ (%)	17.3	16.6	15.0
$R_{free,neutron}$ (%)	18.4	18.0	16.7
**Refinement (X-ray part)**			
Resolution range (Å)	19.2 - 0.99	27.5 - 0.96	27.5 - 0.93
No. of reflections (total / free)	121618 / 6110	132539 / 6648	145639 / 7330
$R_{cryst,X\text{-}ray}$ (%)	9.4	9.3	9.6
$R_{free,X\text{-}ray}$ (%)	10.3	10.4	10.3
**Refinement (general part)**			
No. of refined protein residues	223	223	223
Protein hydrogen atoms visible (%)	90	90	91
No. of ligand non-hydrogen atoms	-	9	9
No. of ligand hydrogen atoms	-	14	9
No. of other ligand atoms [b]	6	6	6
No. of water molecules	177	171	175
No. of visible water hydrogens	242	244	261
RMSD, bond lengths (Å)	0.008	0.007	0.006
RMSD, bond angles (˚)	1.3	1.4	1.2
Ramachandran plot (%) [c]			
Most favored / favored	88.3 / 99.1	88.3 / 99.1	89.4 / 99.1
Additionally allowed / allowed	11.7 / 0.9	11.7 / 0.9	10.6 / 0.9
Generously allowed / -	0.0 / -	0.0 / -	0.0 / -
Disallowed / outliers	0.0 / 0.0	0.0 / 0.0	0.0 / 0.0
Average *B* factors ($Å^2$) [d]			
All protein atoms	20.5	19.4	17.0
Main chain	17.3	16.2	13.9
Side chain	21.6	20.5	18.1
Ligand atoms	-	23.7	17.1
Other ligand atoms [b]	42.3	35.9	24.2
Waters	36.8	35.5	32.7

[a] All crystals used for diffraction experiments have been deuterated before data collection (for details see Experimental Section). Although H and D atoms are distinguished by neutron crystallography, all H and D atoms are referred to as hydrogens in the table.
[b] Other ligands are sulfate and Ca^{2+}
[c] Calculated using PROCHECK[4] and MOLPROBITY[5], respectively; first value indicates PROCHECK results, second MOLPROBITY values as calculated in the PDB validation report
[d] Calculated using MOLEMAN including hydrogen atoms[6]

Supplementary Table 8. Residence times of water molecules for selected amino acids in protein and bulk solvent environment.

	τ_{trans} [ps][a]	$\tau_{trot,X}$ [ps][a]	$\tau_{trot,Y}$ [ps][a]	$\tau_{trot,Z}$ [ps][a]
Tyr228	10.6 ± 3.8	6.16 ± 2.03	7.10 ± 2.40	4.66 ± 1.74
Tyr228 bulk	1.85 ± 0.13	0.94 ± 0.04	0.994 ± 0.045	0.777 ± 0.023
Asp189	87.6 ± 48.7	42.8 ± 24.1	53.8 ± 28.9	24.78 ± 20.88
Asp189 bulk	6.27 ± 0.22	2.20 ± 0.08	2.24 ± 0.09	1.42 ± 0.05
His57	1.68 ± 0.35	1.27 ± 0.20	1.31 ± 0.22	1.14 ± 0.14
His57 bulk	1.37 ± 0.05	1.02 ± 0.03	1.02 ± 0.03	0.925 ± 0.029

[a] All values are given as averages (± 1 s.d.) over 2 ns slices of the full trajectory.

Supplementary Table 9. Damping factors for individual molecular motions, defined as the ratio between the solvent residence time found in the protein **(p)** and the bulk solvent **(b)** environment.

**Amino Acid**	τ_{trans}**(p)/**τ_{trans}**(b)**	$\tau_{rot,X}$**(p)/**$\tau_{rot,X}$**(b)**	$\tau_{rot,Y}$**(p)/**$\tau_{rot,Y}$**(b)**	$\tau_{rot,Z}$**(p)/**$\tau_{rot,Z}$**(b)**
Tyr228	5.73 ± 2.06 [a]	6.58 ± 2.19	7.14 ± 2.43	5.99 ± 2.25
Asp189	14.0 ± 7.8	19.4 ± 11.0	24.1 ± 13.0	17.4 ± 14.7
His57	1.22 ± 0.26	1.25 ± 0.20	1.28 ± 0.22	1.23 ± 0.16

[a] The uncertainties indicate ± 1 s.d. and are obtained from error propagation of the individual quantities as listed in Supplementary Table 1.

Supplementary Table 10. Summary of the data on the comparison between crystallographic and computed water molecules.

**Crystallographic Water**	**$d_{O\text{-}O}$ [Å]**[a]	**$RMSD_{H\text{-}H}$ [Å]**[b]	**Reference frame**
W1 orientation A [c]	2.82 ± 0.65	1.04 ± 0.21	Tyr228
W1 orientation B [d]	2.82 ± 0.65	0.911 ± 0.259	Tyr228
W7	1.27 ± 0.85	0.616 ± 0.184	Tyr228
W2 configuration A [e]	1.62 ± 0.53	0.916 ± 0.224	Asp189
W2 configuration B [f]	2.03 ± 0.83	1.15 ± 0.18	Asp189
W3 orientation A [g]	1.12 ± 0.52	1.11 ± 0.17	Asp189
W3 orientation B [h]	1.12 ± 0.52	1.14 ± 0.16	Asp189

[a] Average distance, $d_{O\text{-}O}$, between crystallographic water oxygen atom and simulated water oxygen atom calculated from the set of first closest water molecules found for each frame during the MD simulation. The closest distances were calculated within a reference frame (last column).
[b] As a simplified measure of the orientation overlap between crystallographic and computed water molecules, the shortest RMSD between hydrogen atoms after superimposing the oxygen atoms was calculated ($RMSD_{H\text{-}H}$).
[c] O-H pointing on Tyr228 aromatic plane
[d] O-H pointing into S_1 pocket
[e] O-H pointing into S_1 pocket
[f] O-H pointing on Ser190
[g] O-H pointing on Asp189-O_δ
[h] O-H pointing on Lys224-O

Supplementary Table 11. Atoms used to define the principal axis vectors and reference frame.

**Side chain**	**z-axis subset** [a]	**x-axis subset**	**z-axis reference**	**1st shell cutoff [Å]**
Tyr228	$C_{\delta 1/2}$, $C_{\varepsilon 1/2}$	C_{γ}, C_{ζ}	Trp215<br>side chain	4.0
Asp189	C_{β}, C_{γ}, $O_{\delta 1/2}$	C_{β}, C_{γ}	Cys191<br>side chain	3.5
His57	C_{γ}, C_{δ}, N_{δ}, C_{ε},<br>N_{ε}	C_{γ}, N_{ε}	Cys58<br>side chain	4.0

[a] Please note that the z-axis is defined as the vector orthogonal to the plane spanned by these atoms.

Supplementary Note 1

Translational and Rotational Stability of W1 water molecules

The W1 water molecule is located on top of the phenyl moiety of the Tyr228 residue in trypsin (same numbering in related proteins such as thrombin). Since it is buried deep within the S_1 pocket and many of the known binders displace or interact with this water molecule, W1 may play an important role for the functionality of the protein. It has been suggested that its low thermodynamic stability makes it beneficial to displace, most likely due to the hydrophobic environment provided by Tyr228. However, little is known about the time scale on which (i) W1 water molecules enter/leave the Tyr228 hydration layer and (ii) W1 water molecules sample different orientations. Therefore, the lifetimes of the translational and orientational states of W1 water molecules during a 0.5 μs MD trajectory of the unbound trypsin structure were investigated. The translational process analyzed herein is the escape from the first hydration layer of Tyr228, which is defined as the space within 4.0 Å of the ring center of the aromatic portion of Tyr228. The investigated orientational process is the loss of orientation of different water reference axes, which are defined internally by the water molecule itself and are explicitly related to the orientation of Tyr228. Auto-correlation functions were used for the calculation of the lifetimes of these processes. Details on the actual computation can be found in the Supplementary Experimental Section.

The average lifetime of the W1 water molecules in the first hydration layer of Tyr228 was estimated to 10.6 ± 3.8 ps (± one s.d.), whereas solvent reorientation occurs on the time-scale of 4.6 to 7.1 ps as indicated by its residence time values (see Supplementary Table 8). Generally, the damping factor, in the following defined as the ratio of the solvent residence time found in the protein environment and the solvent residence time found in bulk solvent, is comparable for each residence time observed for the W1 water molecules (see Supplementary Table 9). In comparison to the crystal structures, the spatial positions of the W1 water molecules

were, on average, not very well reproduced. They were missed by approximately 2.82 Å when only considering the nearest water molecules of the MD simulation (see Supplementary Table 10). As another example, the crystallographic water molecule W7 was reproduced much better with a deviation of only 1.27 Å relative to its crystallographic reference position. Presumably, this water W7 is fixed within the interior of the protein and therefore restrained with respect to translational and orientational degrees of freedom. The orientations of water molecules at W1, however, seem not to favor any of the two crystallographic conformations A or B as suggested by the $RMSD_{H\text{-}H}$ values found for the two conformations (see Supplementary Table 10). This observation is in accordance with the fact that both orientations of the W1 refined to nearly identical occupancy values. On the contrary, water molecules that interact with Asp189, namely W2 and W3, are highly stable in space and have a translational lifetime as long as 87.6 ps. By this, its orientational lifetime is much longer even if compared to the translational lifetime of the W1 water molecules. Furthermore, the damping factor for water molecules next to Asp189 are ranging from 14.0, for the translational damping, to 24.1 for the R_Y vector. The side chain of Asp189 is surrounded on average by 1.7 water molecules during the MD simulation. The position of the crystallographic water molecule W2 was not very well reproduced by MD in both configurations when considering only the nearest water molecule. In contrast, W3 was spatially reproduced very well, (on average 1.12 Å as nearest distance), which is even closer than in the case of W7. However, the orientations are not equivalently reproduced by the simulation. Similarly here, no clear preference is indicated over the two orientations found in the crystal structure. As a reference, His57, an important residue in the catalytic triad of the protein, was investigated. It is located at the surface of the protein and highly accessible by water molecules from the bulk phase. Therefore, we assume, that the dynamic properties of the water molecules interacting with this His57 residue do not differ tremendously with respect to the same residue in a completely solvent-exposed situation. As indicated by the damping factors

of His57, all of them being close to unity, this residue does indeed obey bulk-like solvation dynamics.

Given the high occupancy of almost 75% and fast translational dynamic in the first hydration layer of Tyr228 during the MD simulation, it is obvious that water molecules must rapidly enter and leave the hydration layer. Furthermore, all orientational lifetimes for W1 water molecules are comparable to each other within one standard deviation. Moreover, the fast decay of orientational states matches with the observation of multiple orientational states of W1 in the crystallographic part of this study. On the contrary, water molecules W2 and W3, both attached to Asp189, follow slow exchange dynamics. Given the high occupancy of 96% (1.71 water molecules on average) in the first hydration layer of Asp189, it can be assumed that W2 and W3 exchange indeed slow. With respect to their high damping factors, it can be assumed that the shape and electrostatic distribution of the S_1 pocket takes a significant impact on these water molecules. It is quite remarkable that, although water molecules at W1 position are in fact in hydrogen bonding distance to W2 and W3, they obey completely different dynamics.

Supplementary Note 2

Observation of a planar and pyramidal geometry at the central guanidino nitrogen

Isolated *N*-amidinopiperidine adopts on average a planar conformation during a short QM molecular-dynamics (MD) simulation regardless of whether the pyramidal or planar form was used as a starting point (Supplementary Figure 3A-B in red). In contrast, the respective geometry was on average maintained in the presence of trypsin during short QM/MM simulations further underlining that the formation of a bidentate salt bridge seems to contribute to the stability of the pyramidal piperidine nitrogen (Supplementary Figure 3A-B in blue). A close inspection of all simulation data revealed that formation of the protein-ligand complex does not only permit the existence of the pyramidal *N*-amidinopiperidine form but also causes a lengthening of the bond between the ligand's piperidine nitrogen and the attached amidino carbon by ~ 0.02 Å (Supplementary Figure 3C-D). Similarly, the two cyclic guanidine examples found in the CSD (see above) are also taking part in a bidentate salt bridge and they are characterized by a lengthening of the corresponding CN bond to 1.351 Å ± 0.001 Å, while all other cyclic guanidine molecules lacking such a tight interaction display a significantly shorter mean CN bond length of 1.330 Å ± 0.008 Å. Based on these data, we propose that, upon formation of a bidentate salt bridge, charge is transferred from the carboxylate to the LUMO of *N*-amidinopiperidine (Supplementary Figure 4), which is anti-bonding. This is the likely cause for the observed CN bond lengthening and is associated with a decoupling of the amidino group from the neighboring piperidine system, which facilitates pyramidalization.

Supplementary References

1. Wang W, Gu J, Zou X, Tong W, Gong H. Solid state studies of the assembly of diionic guanidinium/carboxylate compounds. *Tetrahedron Lett* **56**, 2684-2687 (2015).

2. Fukada H, Takahashi K. Enthalpy and heat capacity changes for the proton dissociation of various buffer components in 0.1 M potassium chloride. *Proteins* **33**, 159-166 (1998).

3. Goldberg RN, Kishore N, Lennen RM. Thermodynamic quantities for the ionization reactions of buffers. *J Phys Chem Ref Data* **31**, 231-370 (2002).

4. Laskowski RA, MacArthur MW, Moss DS, Thornton JM. PROCHECK: a program to check the stereochemical quality of protein structures. *J Appl Crystallogr* **26**, 283-291 (1993).

5. Chen VB*, et al.* MolProbity: all-atom structure validation for macromolecular crystallography. *Acta Crystallogr D* **66**, 12-21 (2010).

6. Kleywegt GJ. MOLEMAN - unpublished program. Uppsala University (1992-2004).
