# Supplementary material for: Intriguing role of water in protein-ligand binding studied by neutron crystallography on trypsin complexes
Source: Nat Commun. 2018 Sep 3;9:3559. doi: 10.1038/s41467-018-05769-2 (PMC6120877; doi:10.1038/s41467-018-05769-2)
Supplement: Supplementary file 3 — Description of Additional Supplementary Files [file 41467_2018_5769_MOESM3_ESM.pdf]

Description of Additional Supplementary Files

File Name: Supplementary Movie 1
Description: Exemplary unbinding event of N-amidinopiperidine from trypsin as derived from the corresponding metadynamics trajectory. The movie was generated using VMD and has been smoothed applying a trajectory smoothing window size of 20 ps. It starts at 0.4 ns and ends at 5.0 ns in relation to the full trajectory and plays at a rate of 3.6 ns min-1 . The protein residues Asp189, Ser190, Tyr228 and Gly219 are shown in stick representation lacking non-polar hydrogen atoms, while Namidinopiperidine is represented by an all-atom stick model. Water molecules located in internal cavities at the beginning of the displayed process are represented by individually colored ball-and-stick models. In contrast, water molecules that enter from the bulk water phase during the dissociation event, are displayed as simple sticks.

File Name: Supplementary Movie 2
Description: Exemplary binding event of N-amidinopiperidine to trypsin as derived from the corresponding metadynamics trajectory. The movie was generated using VMD and has been smoothed applying a trajectory smoothing window size of 20 ps. It starts at 87.0 ns and ends at 99.8 ns in relation to the full trajectory and plays at a rate of 9.0 ns min-1 . The protein residues Asp189, Ser190, Tyr228 and Gly219 are shown in stick representation lacking non-polar hydrogen atoms, while N-amidinopiperidine is represented by an all-atom stick model. Water molecules located in internal cavities at the end of the displayed process are represented by individually colored ball-and-stick models. In contrast, water molecules that are released from the binding site upon ligand association, are displayed as simple sticks.

File Name: Supplementary Movie 3
Description: Exemplary unbinding event of benzamidine from trypsin as derived from the corresponding metadynamics trajectory. The movie was generated using VMD and has been smoothed applying a trajectory smoothing window size of 20 ps. It starts at 125.4 ns and ends at 132.8 ns in relation to the full trajectory and plays at a rate of 3.6 ns min-1 . The protein residues Asp189, Ser190, Tyr228 and Gly219 are shown in stick representation lacking non-polar hydrogen atoms, while benzamidine is represented by an all-atom stick model. Water molecules located in internal cavities at the beginning of the displayed process are represented by individually colored ball-and-stick models. In contrast, water molecules that enter from the bulk water phase during the dissociation event, are displayed as simple sticks.
